# Green Synthesis, Characterization, and Empirical Thermal Conductivity Assessment of ZnO Nanofluids for High-Efficiency Heat-Transfer Applications

**DOI:** 10.3390/ma16041542

**Published:** 2023-02-12

**Authors:** Meriem Jebali, Gianpiero Colangelo, Ana Isabel Gómez-Merino

**Affiliations:** 1 Department of Engineering for Innovation, University of Salento, 73100 Lecce, Italy; 2 Department of Applied Physics II, University of Málaga, 29071 Málaga, Spain

**Keywords:** ZnO, thermal conductivity, green synthesis, nanofluids

## Abstract

ZnO nanoparticles were synthesized using lemon juice and zinc nitrate (1:1) through the green method. The structure of the biosynthesized ZnO nanoparticles was analyzed by X-ray diffraction (XRD), Fourier transform infrared spectroscopy (FTIR), and thermogravimetric analysis (TGA). The morphology and the size of ZnO nanoparticles were elucidated by transmission electron microscopy (TEM) and scanning electron microscopy (SEM). The powder was highly dispersed and irregularly shaped and the size of the nanoparticles ranged from 28 to 270 nm, depending on the shape of the particles. Thermal conductivity of the biosynthesized ZnO PG/W mixture 40:60 (*v*/*v*) nanofluids was measured within the temperature range of 20–70 °C. Experimental results revealed a linear increase in thermal conductivity with the rise of temperature and volume fraction. The enhancement of this parameter with temperature was probably due to the different shapes of the former agglomerates. They were broken by the thermal energy in aggregates of different forms. A correlation of these structures with temperature was established. Finally, an empirical model was developed for predicting thermal conductivity with particle volume fraction and temperature.

## 1. Introduction

Water, ethylene glycol, propylene glycol, and oil are the most used conventional heat-transfer fluids in various industries such as power generation, transportation, heating, air-conditioning, biomedical, and ventilation [1,2]; however, these working fluids are poor heat-transfer fluids since their thermal conductivity plays an important role in the convective heat transfer. Miniaturization, enhanced power, and the possibility of increasing the efficiency of devices have encouraged researchers to progress and develop a new generation of fluids, called nanofluids, which are dilute liquid suspensions of nanoparticles with at least one of their main dimensions being less than 100 nm. The motivation for nanofluids can be traced back to Maxwell’s prediction of improving the thermal conductivity of liquids using solid particles [3]. Nanofluids have been found to possess enhanced thermo-physical properties such as thermal conductivity, thermal diffusivity, viscosity, and convective heat-transfer coefficients compared to traditional heat-transfer fluids [4]. Nanofluids might be generated in a single base fluid or a single-phase liquid mixture. The use of propylene glycol/water mixture and ethylene glycol/water mixture in car radiators as a coolant [5,6], industrial heat-exchangers [7], electronic devices [6], and diesel-electric generators [8] prompted investigation of nanofluids’ properties produced with these base fluids [9]. In cases where the base fluid is a mixture of liquids, the base fluid is prepared by mixing the liquids in desired quantities. Nanoparticles of required mass are then dispersed in them for the formulation of nanofluids.

So far, a variety of nanoparticle materials in suspension have been studied, including alumina [10,11,12], zinc oxide [11,12,13,14], titanium dioxide [12,15], copper oxide [5,11,16], ferric oxide [17,18], silica [11,19], graphene [7,20,21], carbon nanotubes [22], and nanodiamond [23]. ZnO nanoparticles and their nanofluids have been chosen in this study because of their thermal behavior [24,25,26,27]. Several methods are used to synthesize zinc oxides nanoparticles (ZnO NPs). These include the co-precipitation method [28], solvothermal method [29], sol-gel method [30], hydrothermal method [31], vapor-transport method [32], and thermal decomposition [33]; nevertheless, most of the identified methods utilized sophisticated equipment, toxic chemicals, and generate environmentally risky by-products; moreover, to produce metallic oxide nanoparticles, harmful chemicals such as oxalic acid, diethylenediamine, sodium borohydride, and hydrazine have been used during the process and react as reducing and capping agents [34]. These poisonous chemicals are not environment-friendly and their presence on the nanoparticles/nanofluids’ surface could increase the toxicity problem. To fix these issues, environmentally friendly and “green” synthesis procedures have been used recently, which are intended to avoid the use of toxic reagents in order to reduce energy consumption, and to use ecological solvents.

In the past decade, utilizing biological systems to synthesize ZnO nanoparticles (ZnO NPs) has been greatly explored and several studies have been focused on plants and their extracted parts as reducing and capping agents for the preparation of ZnO NPs [35,36,37,38]. Several factors could affect the synthesis of ZnO NPS using the green route, including concentration of the precursor, pH of the solution, annealing temperature, and plant-extract concentration. Doan Thi et al. [38] synthesized ZnO NPs using orange fruit peel and addressed the influence of the annealing temperature (from 300 to 900 °C) and the synthesis pH (from 4 to 11) on the size and the shape. O.J. Nava et al. [39] found that the non-similarity in size and shape of the synthesized ZnO NPs depends on the fruit peel extract used during the green synthesis. F. Davar et al. [36] investigated the effect of using different volumes of lemon juice (5–70 mL) on the size distribution of ZnO nanoparticles. N. Ain Samat et al. [40] demonstrated that the size of the biosynthesized ZnO NPs depends on the zinc acetate concentration. All the results mentioned above show that the green synthesis of zinc oxide nanoparticles is eco-friendly, avoiding the utilization of harmful synthetic compounds, is easy, and is a cost-effective method.

In the present paper, ZnO NPs were synthesized through a green route by means of using lemon juice extract and then dispersed in a mixture of propylene glycol and water, using different ultrasonication times, to obtain nanofluids of various concentrations. The structure and morphology of the ZnO NPs were characterized by using X-ray diffraction (XRD), Fourier transform infrared spectroscopy (FTIR), scanning electron microscopy (SEM), transmission electron microscopy (TEM), and thermogravimetric analysis (TGA). The effect of volume particle concentration, stability, and thermal conductivity were investigated. The main objectives of this work were to successfully synthesize and characterize zinc oxide nanoparticles by avoiding the addition of any toxic component to the preparation procedure, to detect an enhancement in the thermal properties of the base fluids in the presence of biosynthesized ZnO nanoparticles, and to develop an empirical model for predicting thermal conductivity with particle volume fraction and temperature.

## 2. Materials and Methods

### 2.1. Chemicals and Reagents

Extra-pure zinc nitrate hexahydrate (Zn (NO_3_)_2_.6H_2_O) was used as zinc precursor and purchased from SRLchem BIOLABO, Tunis, Tunisia. Pure propylene glycol and distilled water were used as base fluid. All glassware was appropriately cleaned with distilled water and then dried.

### 2.2. Preparation of ZnO Nanoparticles

**Figure 1:**
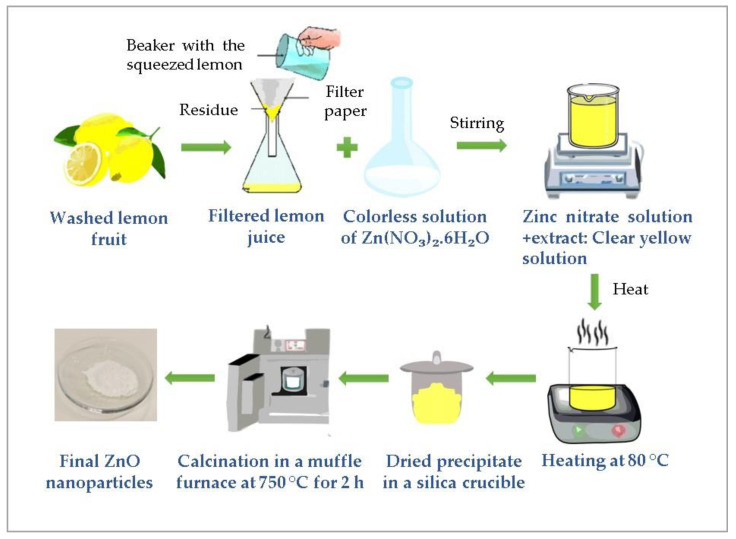
Illustration of the green synthesis process of ZnO nanoparticles using lemon juice.

In this study, ZnO nanoparticles were prepared by the green synthesis route. First, lemon fruits were acquired from a daily market in Tunis. The fresh lemons were washed with distilled water. After air drying at ambient temperature, the lemon fruits were squeezed and the lemon juice was double filtered with a Whatman filter paper. Later, Zn (NO_3_)_2_.6H_2_O was completely dissolved in distilled water under stirring to form an uncolored solution; then, the lemon juice was mixed with the nitrate solution at room temperature by means of a magnetic stirrer (Agimatic N, P-Selecta, Barcelona, Spain). After that, the mixture was heated up to 80 °C under continuous stirring at 500 rpm until a yellow gel was formed. Finally, the gel was transferred to a ceramic crucible (Fisher Scientific SL, Madrid, Spain) followed by calcination in a furnace at 750 °C for 2 h. The ZnO nanoparticles’ synthesis was carried out with a volume ratio between the zinc nitrate solution and the lemon juice of 1:1. The schematic illustration of the green synthesis process of ZnO nanoparticles using lemon juice as an extract plant is represented in Figure 1.

- Mechanism of ZnO formation by green synthesis

**Figure 2:**
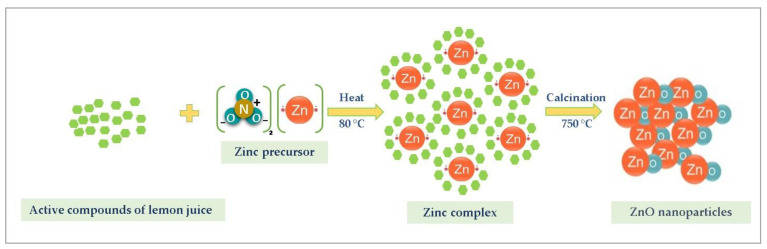
Mechanism of the formation of ZnO nanoparticles.

Green synthesis of nanoparticles using plants has been used the most recently because of the cost-effectiveness, less toxicity, and the rapidity of the vegetal substrates compared to bacteria, algae, and fungus. As it is reported in previous works, the plant extracts are rich in active compounds and are considered as antioxidants, such as phenolic acid, flavonoids, carotenoids, etc. [41]. These active compounds are responsible for the green synthesis of metal oxide nanoparticles due to their ability to act as antioxidant agents and stabilizers during the synthesis of the nanoparticles [42]. Despite the knowledge of the active compounds of the plant extract properties, the precise mechanism of the biosynthesis of metal oxide using the green route is still a challenge to be performed. The chemical composition of Tunisian lemon juice has been investigated by Gargouri et al. [43]. The active compounds present with higher concentrations in the lemon juice and responsible for the ZnO formation are: Quercetin, Naringenin, Limocitrin, Citric acid, Malic acid, Ascorbic acid, Hydroxybenzoic acid, and Hydroxycinnamic acid. These biomolecules may act as stabilizers and/or complexing agents and affect different properties, such as the size of the ZnO nanoparticles; however, no data about the chemical nature of these complexes, which may be chemically altered by heat treatment at 80 °C, have been available up to now. For that reason, we exclude, in principle, reduction of Zn^2+^ ions to metallic zinc, as an intermediate process leading to the formation of ZnO NPs. Figure 2 represents the possible mechanism of the green synthesis process of ZnO nanoparticles using lemon juice which could be divided into three steps: hydrolysis, complexation, and thermal decomposition [44]. During the calcination process, the obtained Zn complex was decomposed to ZnO nanoparticles.

### 2.3. Characterization Techniques

X-ray diffraction (XRD) was investigated by using a D8 Advance Bruker diffractometer with CuKα radiation (λ = 1.5418 Å) from 20° to 80° at a rate of 0.002 2𝜃/s. The obtained diffractogram was processed with the X’pert High Score Plus software 2.1.0. Fourier Transform Infrared (FT-IR) measurements were recorded using a PerkinElmer Frontier MIR/FIR spectrophotometer (Villebon-sur-Yvette, France) in KBr pellets in the spectral range of 400–4000 cm^−1^. Scanning electron microscopy (SEM) was carried out using a Philips XL-30 (20 kV, magnification 31,000× and 70,000) (Eindhoven, Netherlands). Transmission electron microscopy (TEM) was performed using a TEM JEM-1400 (JEOL Company, Massachusetts, USA) with an accelerating voltage of 120 KV. Thermogravimetric analysis (TGA) was conducted using a PerkinElmer STA 6000 thermal analyzer in the range of 25–750 °C with a heating rate of 10 °C/min under nitrogen.

### 2.4. Preparation of ZnO Nanofluids

To prepare different fractions of the nanofluids, the required amounts of mass for the base fluid at 40:60 (PG/W) were first calculated. The ZnO nanofluid was prepared by adding the weighted nanoparticles to the PG solvent followed by the addition of the proper mass of water and mixing with a magnetic stirrer at 700 rpm for 1 h; then, the obtained suspension was exposed to ultrasonic waves for 3 h, which reduced the nanoparticle clusters. The nanofluids were prepared in particle concentrations of 0.5%, 1.0%, and 1.5%. No surfactant was used for the preparation of the suspensions as they may have some influence on the thermal properties of nanofluids.

### 2.5. Stability and Characterizations of Nanofluids

Dispersion characteristics of suspensions were evaluated by visual inspection, particle size measurements, and zeta potential analysis. The nanoparticle suspension stability was measured for 30 min using Turbiscan LabExpert (Formulaction, Toulouse, France). Samples were loaded into cylinder glass tubes (Formulaction, Toulouse, France) and analyzed for 30 min for the entire length of the holder. Particle size and zeta potential measurements were conducted using a Zetasizer Nano ZS device (Malvern Panalytical Ltd, Malvern, UK). The particle concentration to perform the measurements was under 25 mg/mL. The mean dimension of clusters of PG/W-based ZnO nanofluids was estimated utilizing dynamic light scattering (DLS), repeating each measurement three times for each sample.

### 2.6. Measurement of Thermal Conductivity

The hotwire method was used for measuring the thermal conductivity of the nanofluid samples according to the standard ASTM D 2717. The measuring system was System Lambda 01/L (Flucon-PLS Systemtechnik, Barbis, Germany) and it was conducted with a platinum wire (with a diameter of 0.1 mm and a length of 35 mm) and a thermocouple, which are vertically inserted into the nanofluid in a cylindrical cell located in a stable temperature bath. Before the experiments, the device was calibrated with distilled water. All the measurements of the thermal conductivity were repeated three times in temperatures ranging from 20 °C to 70 °C.

## 3. Results and Discussions

### 3.1. XRD Analysis

**Figure 3:**
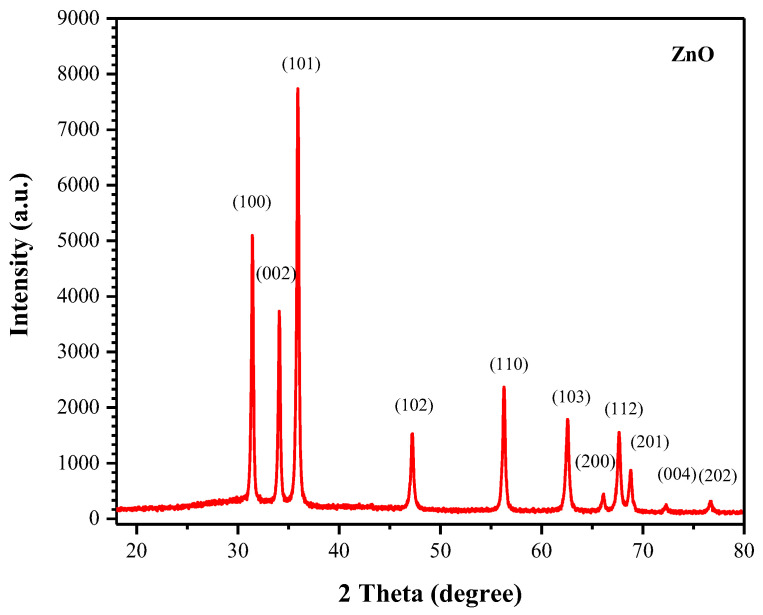
XRD pattern of the biosynthesized ZnO nanoparticles.

Figure 3 shows the diffractogram of the biosynthesized ZnO NPs. The diffraction peaks were observed at positions 2*θ* = 31.425°, 34.084°, 35.912°, 47.213°, 56.275°, 62.553°, 66.081°, 67.645°, 68.783°, 72.278°, and 76.680°, and assigned in terms of the Miller indices (hkl), corresponding, respectively, to the diffraction planes (100), (002), (101), (102), (110), (103), (200), (112), (201), (004), and (202). Pure ZnO crystallizing in a hexagonal wurtzite structure (JCPDS N° 01-079-0208) was observed in this pattern. No additional peaks from impurities were detected, which confirms the high purity of the prepared sample and the efficiency of the lemon juice for synthesizing ZnO NPs without other phases.

The average size of the particles was estimated by using the Scherrer equation [45] which is shown below in Equation (1):

(1) $$D=\frac{K\;\lambda }{\beta \cos\theta }$$

where *D* is the average crystallite size, *K* is the Scherrer constant (0.9), *λ* is the wavelength of X-ray beam used, CuKα (*λ* = 1.5418 Å), *β* is the full width at half maximum intensity of the peak (FWHM), and *θ* is the Bragg’s angle. X-ray diffraction analysis of ZnO NPs was investigated from the angle of 20° to 80°. Based on the calculation, the average crystallite size of ZnO was 30.18 nm.

### 3.2. FTIR Analysis

**Figure 4:**
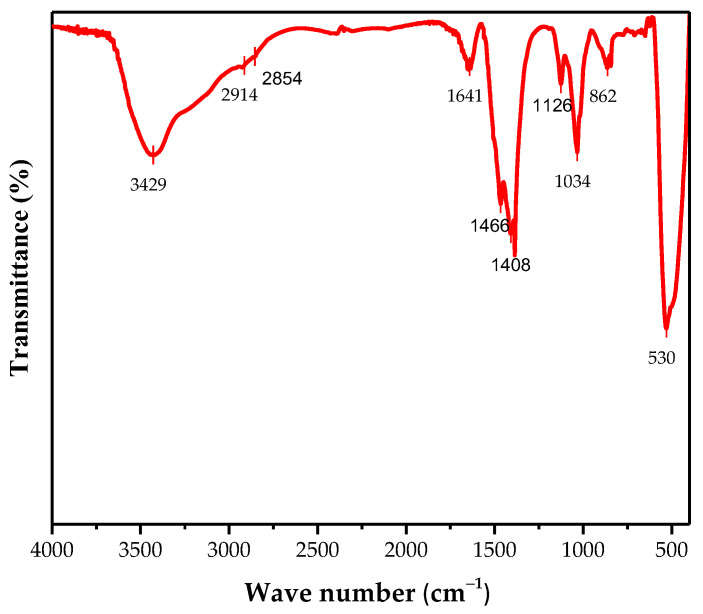
FTIR spectrum of the biosynthesized ZnO nanoparticles calcined at 750 °C.

In FTIR spectrum of ZnO calcinated at 750 °C, shown in Figure 4, the broad absorption band 3428 cm^−1^ was related to the O-H stretching vibration of the adsorbed water [46]. The bands at 2917 and 2854 cm^−1^ were attributed to C-H stretching [47]. The band observed at 1641 cm^−1^ was attributed to be the C=O stretching of the carboxyl group [48]. The bands at 1465 cm^−1^ and 1407 cm^−1^ were due to the asymmetry and symmetry vibration of the -OOH group. The band shown at 862 cm^−1^ could be due to the C-O band [36]. Meanwhile, the bands at 1126 cm^−1^ and 1033 cm^−1^ were related to the aromatic rings and their functional groups of the active compounds of the lemon juice [37]. The presence of the carboxyl group in the ZnO nanoparticles indicated the role of protein as a stabilizer during the crystal growth. The characteristic band corresponding to the Zn-O stretching mode, observed at 530 cm^−1^, confirmed the formation of ZnO particles [38]. The FTIR study showed the possibility of some amount of organic residue from the active compounds of lemon juice in the ZnO sample. The existence of the flavonoids and polyphenols after calcination could be explained by the formation of a strong bond with the zinc (Zn), which creates a layer to protect the particles from agglomeration during the complexation step. These results are consistent with other previous works [36,42].

### 3.3. Morphological Studies

**Figure 5:**
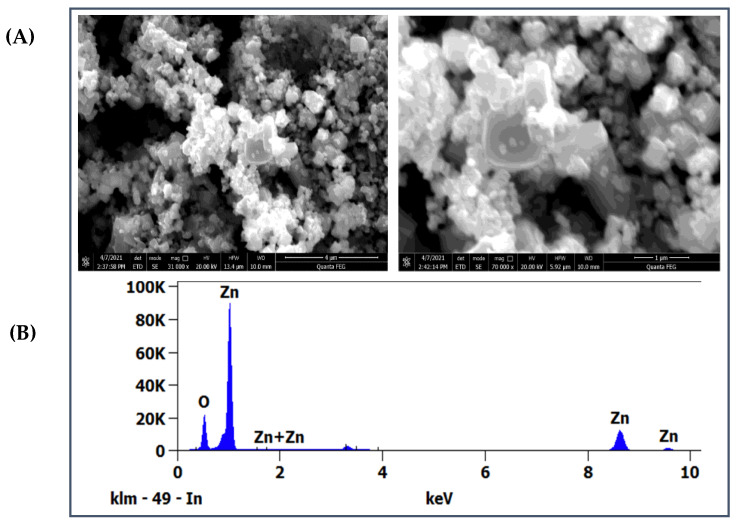
(**A**) SEM micrographs pf the biosynthesized ZnO nanoparticles; (**B**) EDX spectrum.

The SEM micrograph was performed to establish the morphology and the agglomeration nature of the biosynthesized ZnO NPs. As can be seen in Figure 5A, the morphology of the prepared ZnO NPs shows polyhedral shapes and some were irregular and agglomerated. According to Surendra et al. [49], having an agglomerated morphology could be due to the combustion reactions involved during the synthesis. The results of SEM coincided with several previous works using the green method for synthesizing ZnO NPs [35,39]. Chemical purity and composition throughout the biosynthesized ZnO NPs were provided by EDX analysis. Figure 5B exhibits EDX spectrum of ZnO NPs and revealed strong peaks related to Zn and O, confirming the formation of zinc oxide.

**Figure 6:**
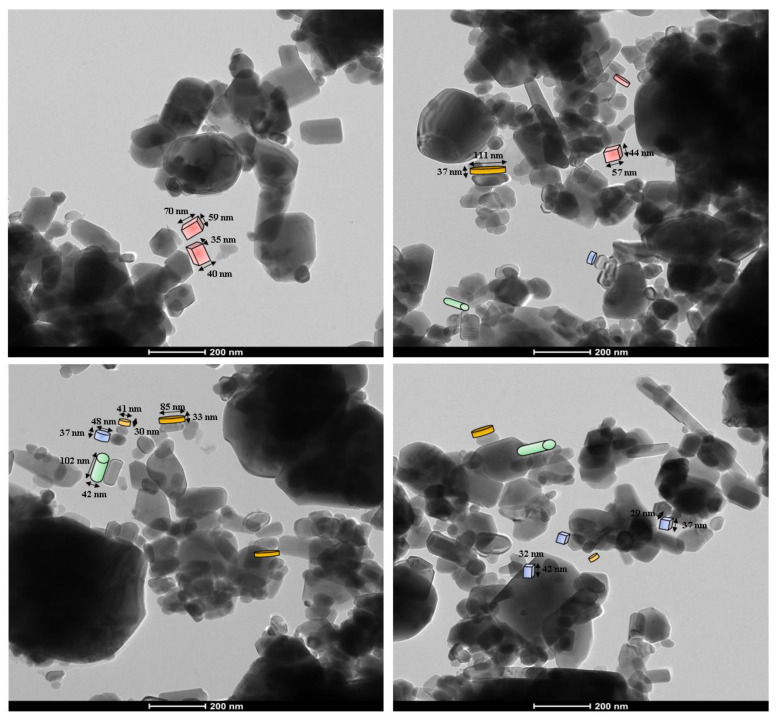
TEM images of the biosynthesized ZnO nanoparticles.

The morphology and the size of ZnO NPs prepared by green synthesis were elucidated by TEM analysis. Figure 6 illustrates that the particles were highly dispersed and with different shapes: platelets, bricks, cylinders, and blades. As it can be seen in the TEM images, the size of the nanoparticles ranged from 28 to 270 nm, depending on the shape of the particle. The size of the particles was calculated using ImageJ software [50].

### 3.4. TGA/DTA Analysis

**Figure 7:**
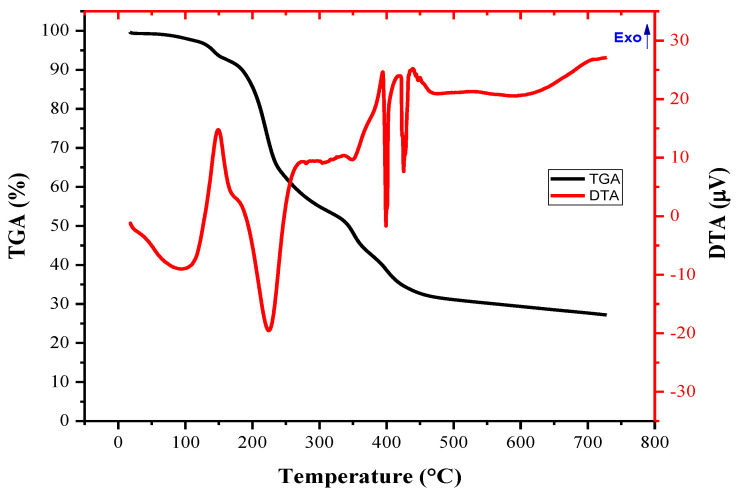
TGA/DTA curve pf the as-prepared ZnO.

DTA/TGA curve of the as-prepared ZnO before calcination is given in Figure 7 at a heating rate of 10 °C/min in nitrogen in a temperature range of 25–750 °C. TGA analysis of ZnO indicated that the first mass loss step (~1.39%) occurred before 187.5 °C, probably due to the vaporization of absorbed water and dehydration reaction. The second mass loss during the heating step (~0.2%) from 187.5 to 350 °C can be associated with the removal of chemisorbed hydroxyl groups. The third step (~0.4%) occurred between 350 °C and 512 °C, which can be related to the oxidation process of the residual organic materials. No further mass loss was observed up to 512.5 °C until 750 °C.

### 3.5. Stability and Characterization of Nanofluid

#### 3.5.1. Stability of Nanofluid

**Figure 8:**
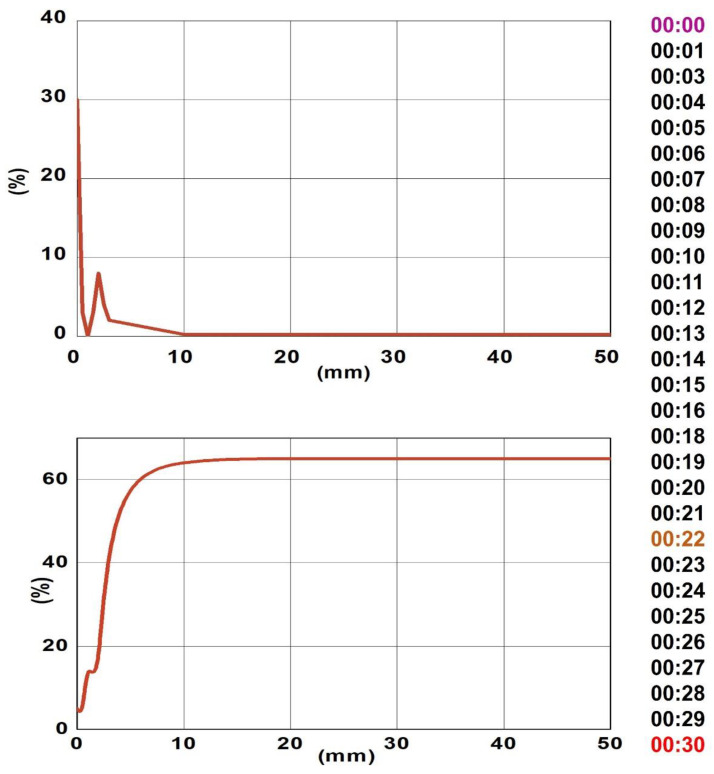
Delta backscattering measurements of 40:60 PG/W-based ZnO nanofluids 1.5 vol%.

The stability of nanofluids has an essential role in their behavior. It becomes basic to investigate stability due to agglomeration and sedimentation. Transmittance and backscattering variations were carried out using a light source in the near-infrared zone at a wavelength of 880 nm. Figure 8 shows the comparison of the delta backscattering values obtained along the cylindrical cell for the nanofluid 1.5 vol%. The backscattering value is straightforwardly proportional to the particle concentration at each position, and it is conceivable to see an increase in backscattering on the lower part of the cell during the measurements. Figure 8 shows that the nanofluid at high concentration 1.5 vol% was stable and no incidence of sedimentation was observed. The same result was observed for the nanofluids at lower concentration 0.5 vol% and 1.0 vol%.

#### 3.5.2. Particle Size Analysis

**Figure 9:**
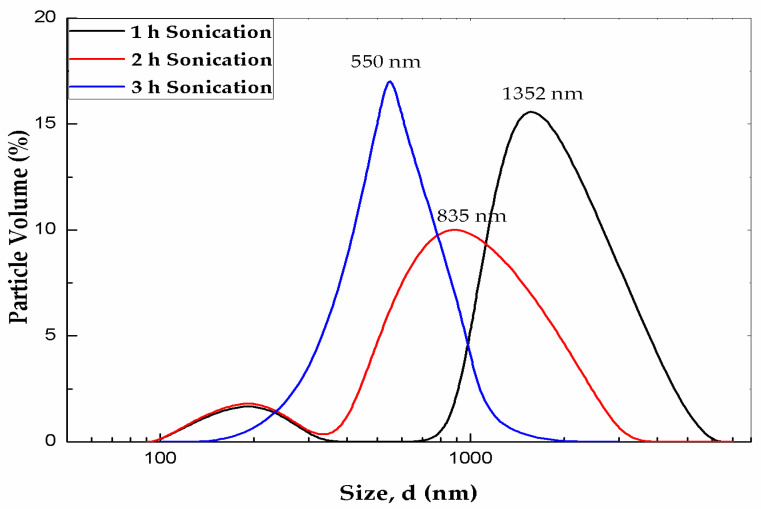
Size distribution of 1.5 vol% ZnO nanofluid dispersed in 40:60 PG/W at different ultrasonication time: 1 h, 2 h, and 3 h. In all measurements, the polydispersity index is 0.4.

Figure 9 shows the particle size distribution of ZnO nanofluids. The average hydrodynamic size was found to be 550 nm and the polydispersity index was 0.4 in all measurements. The hydrodynamic size distribution was larger compared to the results obtained by SEM and TEM analyses. Ghadimi et al. [51] revealed that the cluster of nanofluids would be at least three times higher than the average particle diameter. Ultrasonication is a process of applying frequencies more than 20 kHz to break the clusters of the nanofluids to form a dispersed colloidal suspension [52]. The ultrasonication duration was optimized through the measurement of the average aggregate size variation at different time intervals. A comparison of the particle size distribution analysis of PG/W-based ZnO nanofluids is provided in Figure 9. As indicated before, the agglomerated size decreased with increasing ultrasonication duration from 1 h to 3 h. It can be observed that the size of the clusters is smaller after 3 h of ultrasonication. Since ZnO nanoparticles are bound to interact with water molecules, the presence of larger aggregates was observed.

#### 3.5.3. Zeta Potential Analysis

Zeta (*ζ*) potential is an important basis for measuring the dispersion stability of the surface charge on the particle, also known as electrokinetic potential [53]. The suspension with a ζ-potential value equal or higher than 15 mV is considered a stable colloid. In the present work, the zeta potential values of the nanofluids were about 25.5 ± 0.2 mV [53,54].

### 3.6. Thermal Conductivity

**Figure 10:**
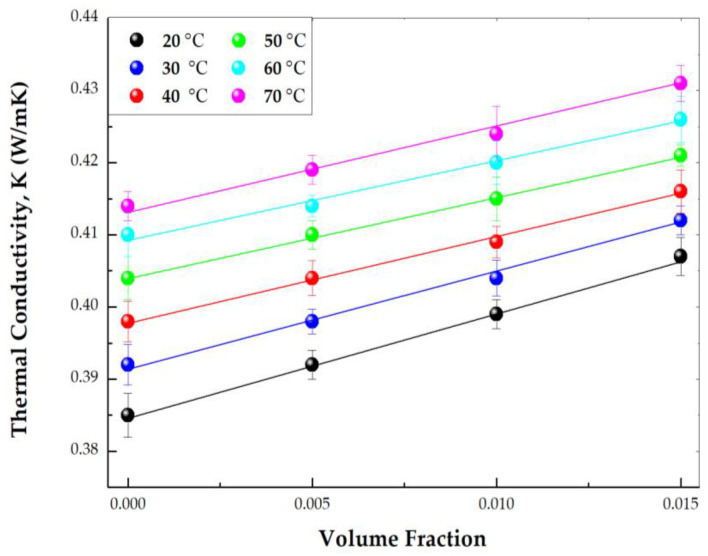
Experimental thermal conductivity versus particle volume fraction of ZnO in 40:60 PG/W nanofluids. Temperature effect.

Figure 10 shows the thermal conductivity vs. volume fraction in the temperature interval of 20–70 °C. In all cases, a linear increase in the thermal conductivity with the ZnO nanoparticles volume concentration was observed. This parameter also increased with temperature, as it has been reported by Bakthavatchalam et al. [55]. The effect of particle concentration on thermal conductivity was more noticeable at lower temperatures. Figure 10 shows a slight increase in the slopes at lower temperatures. As an example, the thermal conductivity enhancements of 0.5 vol%, 1.0 vol%, and 1.5 vol% of 40:60 PG/W-based ZnO nanofluids were 2.85%, 3.63%, and 5.71%, respectively, at 20 °C compared to 40:60 PG/W-based fluid. The linear dependence of the thermal conductivity with particle concentration can be written in Equation (2) as:

(2) $$\frac{k_{nf}}{k_{0}}=1+C_{k}\emptyset ,$$

In this equation, *k_nf_*/*k*_0_ ratio is defined as the thermal conductivity enhancement, *k_nf_* is the thermal conductivity of the nanofluid, and *k*_0_ is the thermal conductivity of the base liquid (40:60 PG/W). *C_k_* is the thermal conductivity enhancement coefficient, which is normalized to the thermal conductivity of the base fluid and *ϕ* is the particle volume fraction. *C_k_* is independent of volume fraction but could change with temperature. This could also be deduced from the change of the slopes with temperature presented in Figure 10. Thermal conductivity can be influenced by many factors such as concentration, type, shape, and size of nanoparticles, temperature, and type of base fluid [56]. Assuming a linear relationship of both particle shape and interfacial contributions with particle volume fraction, thermal conductivity of nanofluids can be described in Equation (3) as follows:

(3) $$\frac{k_{nf}}{k_{0}}=1+\left(C_{k}^{shape}+C_{k}^{surface}\;\right)\emptyset ,$$

where *C_k_^shape^* and *C_k_^surface^* are coefficients related to the contributions to the effective thermal conductivity due to particle shape (positive) and owing to surface resistance (negative), respectively.

**Figure 11:**
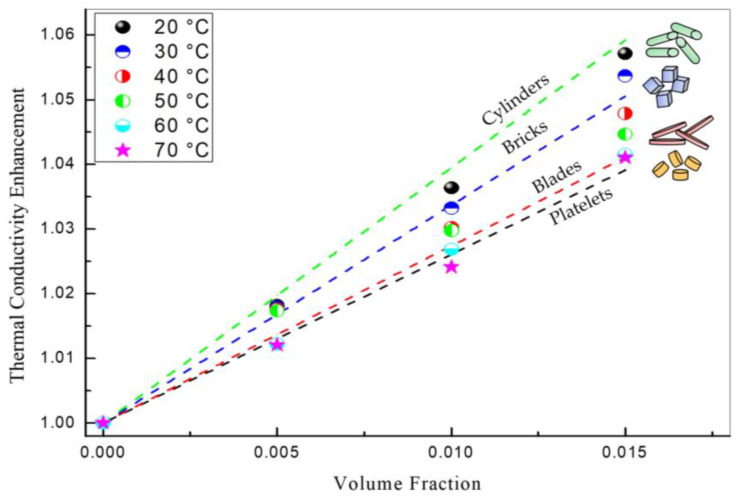
Thermal conductivity enhancement vs. volume fraction. The different shapes are outlined from the TEM microscopy of the powder shown in Figure 5. In this figure, brick, cylinders, blades, and platelets of different sizes are displayed.

The response of these particle shapes to thermal conductivity was studied by different researchers. Timofeeva et al. [57] investigated the effect that nanoparticle shape exerts on viscosity and thermal conductivity for 50:50 EG/W-based suspensions of alumina nanoparticles. They correlated experimental data with particle morphology and obtained the *C_k_* coefficients for the different shapes: platelets, blades, cylinders, and bricks. Figure 11 shows the thermal conductivity enhancement against the volume fraction exhibited in Equation (2). The slopes of the four dash lines correspond with the *C_k_* coefficients obtained for the shapes—cylinders, bricks, blades, and platelets [57]. The experimental values of Figure 11 represent the thermal conductivity enhancement of ZnO NPs. Table 1 presents the experimental slopes, *C_k_*, calculated from the experimental values of Figure 10. As is shown in the TEM images (Figure 6), the ZnO powder is formed by aggregates of several shapes: cylinder, brick, blades, and platelets of different sizes. The column “shape” of Table 1 is obtained through the proportional contribution of the shapes described in [57], according to the values of the slopes obtained for these shapes: platelets (P) 2.61; blades (Bl) 2.74; bricks (Br) 3.37; and cylinders (C) 3.95. As is shown in Figure 6, the TEM images of the ZnO powder exhibit four distinct shapes—bricks, cylinders, blades, and platelets of different sizes—apart from some aggregates. It could be expected that a polydispersity of sizes and shapes could affect the thermal conductivity enhancement. If thermal energy can break aggregates, the increase in temperature would change the shapes and sizes of particles and the thermal conductivity enhancement would also be affected.

For low-concentrated suspensions, it can be assumed that the contribution of interfacial effects should be proportional to the volume fraction and to the particle surface area. The surface factor, *f*, is defined as the ratio between the surface and the volume for a particle of a certain shape and size. It is related to the *C_k_^surface^* in Equation (4) as:

(4) $$C_{k}^{surface}=-fl_{k}$$

Interactions between particles and base fluid are expressed through the interfacial resistance, Kapitza resistance (*R_k_*) at the junction of two materials, which arises due to the chemical and physical (including acoustic mismatch) properties of the two compounds [58]. This parameter is a measure of resistance to heat flow at the interface of two materials and diminishes the entire thermal conductivity of the system. A more operational definition of *R_k_* can be expressed by defining the Kapitza length, *l_k_*, in the following Equation (5):

(5) $$l_{k}=R_{k}k_{0}$$

where *k*_0_ is the thermal conductivity of the base fluid, and *l_k_* is the thickness equivalent to the interface or the thermal resistance length. In the case of normal liquids in contact with a solid, however, it can be expected that *l_k_* should be of the order of a few molecular sizes. For ZnO particles, *k*_0_ is about 0.84 W/mK, in the range of temperatures and particle sizes of this investigation [59]. Timofeeva et al. [57] have estimated the values of the Kapitza length, *l_k_*, and interfacial surface resistance, *R_k_*, within the lower range reported for nanofluids [60]. For ZnO NPs the values of *R_k_* and *f* were evaluated from the values reported in [57] with the corresponding percentage of each shape according to the experimental values of *C_k_* at every temperature. The values of *l_k_*, $C_{k}^{surface}$, and $C_{k}^{shape}$ were evaluated from Equations (3)–(5), respectively. All these values are also shown in Table 1. According to Figure 11 and Table 1, the thermal conductivity enhancement is higher at lower temperatures because the negative contribution of the interfacial resistance $C_{k}^{surface}$, proportional to Kapitza length, is smaller. At higher temperatures, the total surface area of nanoparticles and $C_{k}^{shape}$ (blades and platelets as main shapes) increased but also the negative effect of the interfacial resistance contribution represented as $C_{k}^{surface}$ and the thermal conductivity enhancement, *C_k_*, diminished at higher temperatures. Another interface effect that could enhance thermal conductivity is the formation of a liquid layering at the solid interface. It is supposed that the atomic structure of the liquid layer is considerably more ordered than that of bulk liquid [61]. The preparation of ZnO-Propylene glycol dispersion followed by the addition of water serves to preserve the layers of propylene glycol around the ZnO nanoparticle surface. As Suganthi and Rajan [13] reported in their work, at lower temperatures, the thickness of the ordered arrangement of liquid molecules over nanoparticle surface (called liquid layering) rises leading to an increase in thermal conductivity at lower temperatures for ZnO-propylene glycol dispersion.

**Figure 12:**
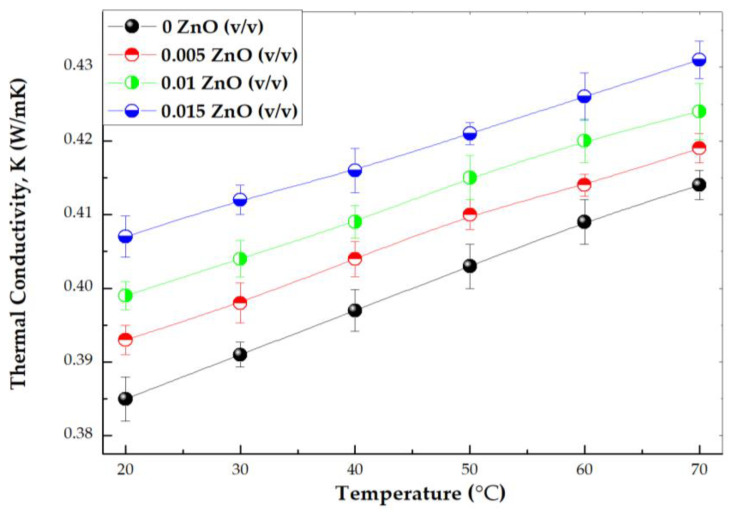
Experimental thermal conductivity vs. temperature of 40:60 PG/W-based ZnO nanofluids.

Figure 12 graphs the experimental thermal conductivity against temperature. For all volume fractions the thermal conductivity linearly boosts with the rise of temperature. This growth of thermal conductivity with temperature could be explained considering the effect of thermal energy on the Brownian movement of nanoparticles at molecular and nanoparticle levels, which has been found to be a key mechanism for the thermal behavior of nanofluids. The increase in nanofluid thermal conductivity is mainly due to the convection induced by the particle Brownian motion, which increases with temperature [55].

**Figure 13:**
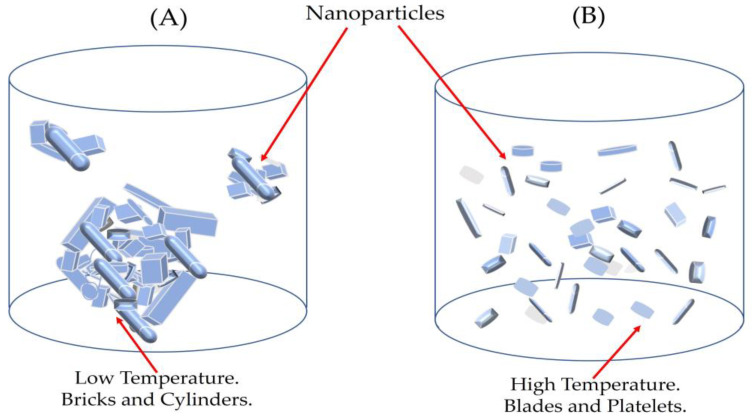
(**A**) At low temperatures, bricks and cylinders are the predominant shapes. They form clusters which could be surrounded by a liquid layer; (**B**) At higher temperatures, broken aggregates of blade and platelet shapes are surrounded by liquid layering, enhancing thermal conductivity.

As it was demonstrated in Figure 11, the growth of temperature reduced the size of particles and the possibility of increasing the total surface area. The TEM picture of the powder (Figure 6) showed agglomeration of particles; however, the DLS measurements revealed the existence of nanoparticles around 500 nm of diameter after 3 h of sonication (Figure 9). According to Table 1, the increase in temperature has led to a breakage of clusters, giving rise to smaller particles of platelet and blade-like shapes. This was quantitatively measured by means of the thermal conductivity enhancement coefficient, *C_k_*, from the slopes of the linear relationship between the relative thermal conductivity and volume fraction. The increase in the total surface area produced by temperature is represented in Figure 13. At low temperatures, particle sizes were bigger and they formed cylinder and brick agglomerates. As temperature increased, these particles reduced in size and changed in shape towards platelets and blades as a consequence of the cluster breakages. Particle agglomerates could also be separated by liquid layers thin enough to allow heat flow among particles. This explanation could justify the thermal conductivity enhancement at lower temperatures.

Table 1 shows how the thermal enhancement coefficient, *C_k_*, varies with temperature, albeit independent of particle volume fraction. An adjustment of these experimental values to a polynomial function together with Equation (2) provided an experimental thermal conductivity model as follows:

(6) $$\frac{k_{eff}}{k_{0}}=1+\left(3.617T^{2}-0.055T+4.77\right)\emptyset$$

The adjustment coefficient, *R*^2^, was 0.98469.

## 4. Conclusions

ZnO nanoparticles were successfully synthesized via a green method and confirmed through X-ray diffraction (XRD), Fourier transform infrared spectroscopy (FTIR), scanning electron microscopy (SEM), transmission electron microscopy (TEM), and thermogravimetric analysis (TGA). The XRD results showed that pure hexagonal phase ZnO powders were formed using this method. The SEM and TEM analyses demonstrated that ZnO powder was formed by aggregates of several shapes, cylinders, bricks, blades, and platelets of different sizes. ZnO propylene glycol/water nanofluids were prepared by the addition of water to ZnO-propylene glycol dispersion. An experimental study was conducted to investigate the effect of particle volume fraction and temperature on the thermal conductivity of PG/W-based ZnO nanofluids. These results revealed that, at low volume fractions, thermal conductivity linearly increased with the raising of temperature and volume fraction; however, the thermal conductivity enhancement, defined as the effective thermal conductivity with respect to the base liquid thermal conductivity, augmented at lower temperatures because the negative contribution of the interfacial resistance was smaller. The nanofluid preparation method serves to preserve the layers of propylene glycol on the ZnO nanoparticle surface, leading to thermal conductivity enhancement at lower temperatures due to liquid layering.

## Figures and Tables

**Table 1:** Contribution of particle shape effect and surface resistance to the thermal conductivity of ZnO powder in 40:60 PG/W.

Temperature (°C)	*C_k_*	$C_{k}^{shape}$	$C_{k}^{surface}$	*f* (nm^−1^)	*l_k_* (nm)	*R_k_* (m^2^ K/W)	Shape
20	3.79 ± 0.09	5.68	−1.89	0.475	3.97	4.727 × 10^−9^	0.73C + 0.27Br
30	3.53 ± 0.13	4.71	−1.18	0.295	4.01	4.773 × 10^−9^	0.27C + 0.73Br
40	3.12 ± 0.14	7.35	−4.23	1.089	3.89	4.626 × 10^−9^	0.71Br + 0.29Bl
50	2.93 ± 0.13	8.67	−5.47	1.43	3.83	4.56 × 10^−9^	0.6Br + 0.4Bl
60	2.79 ± 0.08	13.65	−10.86	3.042	3.57	4.248 × 10^−9^	0.08Br + 0.92Bl
70	2.69 ± 0.14	12.39	−9.70	2.862	3.39	4.04 × 10^−9^	0.6Bl + 0.4P

## Data Availability

Not applicable.

## Nomenclature

D	average crystallite size (m)
W	mass (kg)
K	Scherrer constant
nm	nanometer
T	temperature (°C)
R^2^	adjustment coefficient
*Greek symbols*	
λ	wavelength (m)
𝜌	density (kg/m^3^)
𝜙	volume concentration (%)
𝛽	full width at half maximum intensity of the peak (m)
θ	Bragg’s angle
ζ	zeta potential (mV)
*k*_0_	thermal conductivity of base fluid (W/m·K)
*k_nf_*	thermal conductivity of nanofluid (W/m·K)
*C_k_*	thermal conductivity enhancement coefficient
*R_k_*	Kapitza resistance (m^2^·K/W)
*l_k_*	thermal resistance length (nm)
*f*	surface particle/volume particle
*Subscripts*	
PG	propylene glycol
W	water
NPs	nanoparticles
bf	base fluid
Zn^2+^	zinc ion
P	platelets
Br	bricks
Bl	blades
C	cylinders
